# Crystal structure of bis­[1-(4-bromo­benz­yl)pyridinium] bis­(1,2-di­cyano­ethene-1,2-di­thiol­ato-κ^2^ *S*,*S*′)nickelate(II)

**DOI:** 10.1107/S1600536814024222

**Published:** 2014-11-12

**Authors:** Dong Zeng, Shui-Bin Yang, Zheng-Fang Tian

**Affiliations:** a Hubei Key Laboratory for Processing and Application of, Catalytic Materials, College of Chemical Engineering, Huanggang Normal University, Huanggang 438000, People’s Republic of China

**Keywords:** crystal structure, 1-(4-bromo­benz­yl)pyridinium cation, maleo­nitrile­dithiol­ate dianion, square-planar bis-1,2-di­thiol­ate complex, Ni^2+^ ion, hydrogen bonding

## Abstract

The asymmetric unit of the title salt, (C_12_H_11_BrN)_2_[Ni(C_4_N_2_S_2_)_2_], consists of one 1-(4-bromo­benz­yl)pyridinium cation and one half of a complex [Ni(mnt)_2_]^2−^ (mnt^2−^ is the maleo­nitrile­dithiol­ate dianion). The Ni^2+^ ion is located on an inversion centre and is coordinated by four S atoms from two mnt^2−^ ligands, exhibiting a square-planar coordination environment. In the cation, the planes of the pyridinium and benzene rings make a dihedral angle of 69.86 (19)°. The cations and anions are alternately arranged in layers parallel to (001) and are held together by non-classical C—H⋯N hydrogen bonds.

## Related literature   

For general background to square-planar bis-1,2-di­thiol­ate complexes of transition metals showing potential application as magnetic materials and conductors besides others, see: Duan *et al.* (2010 ▶); Pei *et al.* (2011 ▶); Ren *et al.* (2002 ▶). For the structure of a closely related compound, see: Zhang *et al.* (2011 ▶). For synthetic aspects, see: Davison & Holm (1967 ▶).
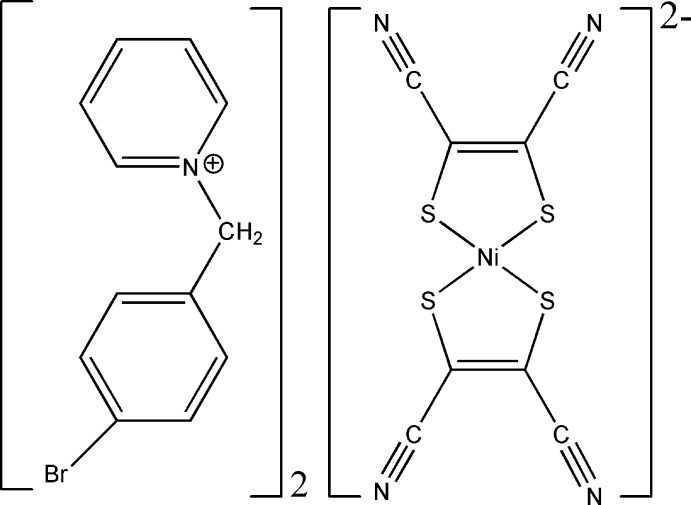


## Experimental   

### Crystal data  

(C_12_H_11_BrN)_2_[Ni(C_4_N_2_S_2_)_2_]
*M* *_r_* = 837.29
Monoclinic, 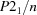

*a* = 9.783 (2) Å
*b* = 11.962 (3) Å
*c* = 14.858 (3) Å
β = 97.385 (7)°
*V* = 1724.3 (6) Å^3^
*Z* = 2
Mo *K*α radiation
μ = 3.16 mm^−1^
*T* = 296 K
0.20 × 0.15 × 0.15 mm

### Data collection  

Bruker SMART CCD diffractometer
Absorption correction: multi-scan (*SADABS*; Bruker, 2000 ▶) *T*_min_ = 0.571, *T*_max_ = 0.649
14683 measured reflections
3040 independent reflections
2204 reflections with *I* > 2σ(*I*)
*R*_int_ = 0.061

### Refinement  

*R*[*F* ^2^ > 2σ(*F* ^2^)] = 0.042
*wR*(*F* ^2^) = 0.102
*S* = 1.03
3040 reflections
205 parameters
H-atom parameters constrained
Δρ_max_ = 0.44 e Å^−3^
Δρ_min_ = −0.91 e Å^−3^

### 

Data collection: *SMART* (Bruker, 2000 ▶); cell refinement: *SAINT* (Bruker, 2000 ▶); data reduction: *SAINT*; program(s) used to solve structure: *SHELXS97* (Sheldrick, 2008 ▶); program(s) used to refine structure: *SHELXL97* (Sheldrick, 2008 ▶); molecular graphics: *SHELXTL* (Sheldrick, 2008 ▶); software used to prepare material for publication: *SHELXL97*.

## Supplementary Material

Crystal structure: contains datablock(s) I, 111. DOI: 10.1107/S1600536814024222/wm5078sup1.cif

**Fig. 1.**
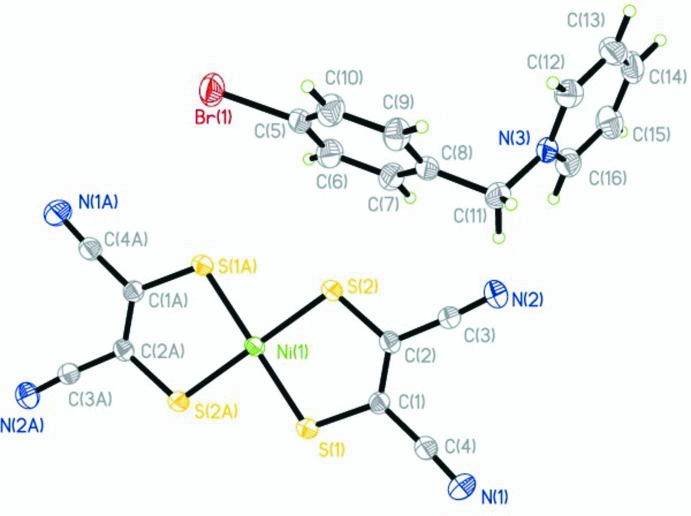
The molecular components of the title structure. Displacement ellipsoids are drawn at the 30% probability level. [Symmetry code: (A) = 1 - x, 1 - y, -z).]

Structure factors: contains datablock(s) I. DOI: 10.1107/S1600536814024222/wm5078Isup2.hkl

x y z . DOI: 10.1107/S1600536814024222/wm5078fig1.tif
The mol­ecular components of the title structure. Displacement ellipsoids are drawn at the 30% probability level. [Symmetry code: (A) = 1 − *x*, 1 − *y*, −*z*).]

**Fig. 2.**
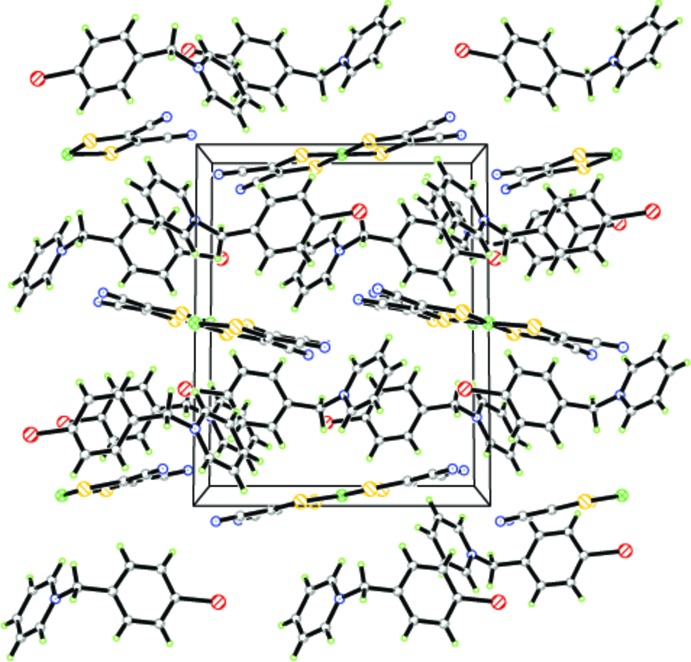
Packing diagram of the title structure viewed along [100]. The origin is at the upper right corner of the unit cell.

. DOI: 10.1107/S1600536814024222/wm5078fig2.tif
Packing diagram of the title structure viewed along [100]. The origin is at the upper right corner of the unit cell.

CCDC reference: 1032163

Additional supporting information:  crystallographic information; 3D view; checkCIF report

## Figures and Tables

**Table 1:** Hydrogen-bond geometry (, )

*D*H*A*	*D*H	H*A*	*D* *A*	*D*H*A*
C16H16N2	0.93	2.49	3.384(6)	162

## supplementary crystallographic information

### S1. Experimental

Disodium maleonitriledithiolate (456 mg, 2.5 mmol) and nickel chloride hexahydrate (297 mg, 1.25 mmol) were mixed under stirring in water (20 ml) and heated to boiling for about 20 min. The resuting red solution was filtered and to the filtrate was added dropwise to an aqueous solution of 1-(4'-bromobenzyl)pyridinium chloride (711.5 mg, 2.5 mmol). The dark red precipitate was filtered off, washed with water three times and dried in vacuum. The crude product was recrystallized from acetone to give red crystals (yield: 76%) with a block-like form.

### S2. Refinement

The H atoms were placed on idealized positions (C—H = 0.93 Å for aromatic and 0.97 Å for methylene H atoms) and refined as riding atoms, with *U*_iso_(H) = 1.2*U*_eq_(C).

### Crystal data

**Table:**

(C_12_H_11_BrN)_2_[Ni(C_4_N_2_S_2_)_2_]	*F*(000) = 836
*M**_r_* = 837.29	*D*_x_ = 1.613 Mg m^−^^3^
Monoclinic, *P*2_1_/*n*	Melting point: 473 K
Hall symbol: -P 2yn	Mo *K*α radiation, λ = 0.71073 Å
*a* = 9.783 (2) Å	Cell parameters from 14683 reflections
*b* = 11.962 (3) Å	θ = 2.2–25.0°
*c* = 14.858 (3) Å	µ = 3.16 mm^−^^1^
β = 97.385 (7)°	*T* = 296 K
*V* = 1724.3 (6) Å^3^	Block, red
*Z* = 2	0.20 × 0.15 × 0.15 mm

### Data collection

**Table:**

Bruker SMART CCD diffractometer	3040 independent reflections
Radiation source: fine-focus sealed tube	2204 reflections with *I* > 2σ(*I*)
Graphite monochromator	*R*_int_ = 0.061
phi and ω scans	θ_max_ = 25.0°, θ_min_ = 2.2°
Absorption correction: multi-scan (*SADABS*; Bruker, 2000)	*h* = −11→11
*T*_min_ = 0.571, *T*_max_ = 0.649	*k* = −14→14
14683 measured reflections	*l* = −17→17

### Refinement

**Table:**

Refinement on *F*^2^	Primary atom site location: structure-invariant direct methods
Least-squares matrix: full	Secondary atom site location: difference Fourier map
*R*[*F*^2^ > 2σ(*F*^2^)] = 0.042	Hydrogen site location: inferred from neighbouring sites
*wR*(*F*^2^) = 0.102	H-atom parameters constrained
*S* = 1.03	*w* = 1/[σ^2^(*F*_o_^2^) + (0.0517*P*)^2^ + 0.2393*P*] where *P* = (*F*_o_^2^ + 2*F*_c_^2^)/3
3040 reflections	(Δ/σ)_max_ < 0.001
205 parameters	Δρ_max_ = 0.44 e Å^−^^3^
0 restraints	Δρ_min_ = −0.91 e Å^−^^3^

### Special details

**Table:**

Geometry. All e.s.d.'s (except the e.s.d. in the dihedral angle between two l.s. planes) are estimated using the full covariance matrix. The cell e.s.d.'s are taken into account individually in the estimation of e.s.d.'s in distances, angles and torsion angles; correlations between e.s.d.'s in cell parameters are only used when they are defined by crystal symmetry. An approximate (isotropic) treatment of cell e.s.d.'s is used for estimating e.s.d.'s involving l.s. planes.
Refinement. Refinement of *F*^2^ against ALL reflections. The weighted *R*-factor *wR* and goodness of fit *S* are based on *F*^2^, conventional *R*-factors *R* are based on *F*, with *F* set to zero for negative *F*^2^. The threshold expression of *F*^2^ > σ(*F*^2^) is used only for calculating *R*-factors(gt) *etc*. and is not relevant to the choice of reflections for refinement. *R*-factors based on *F*^2^ are statistically about twice as large as those based on *F*, and *R*- factors based on ALL data will be even larger.

### Fractional atomic coordinates and isotropic or equivalent isotropic displacement parameters (Å^2^)

**Table:**

	*x*	*y*	*z*	*U*_iso_*/*U*_eq_	
Ni1	0.5000	0.5000	0.0000	0.03497 (19)	
Br1	−0.05497 (5)	0.44064 (4)	0.19128 (4)	0.0857 (2)	
S1	0.68740 (9)	0.59888 (8)	0.02861 (6)	0.0445 (3)	
S2	0.37034 (9)	0.64490 (8)	0.01000 (7)	0.0479 (3)	
C3	0.4306 (4)	0.8595 (3)	0.0544 (3)	0.0472 (9)	
N2	0.3771 (4)	0.9425 (3)	0.0661 (3)	0.0651 (10)	
C8	0.1115 (3)	0.8032 (3)	0.2369 (3)	0.0438 (9)	
C1	0.6257 (3)	0.7321 (3)	0.0478 (2)	0.0398 (8)	
N3	0.0716 (3)	1.0079 (2)	0.2122 (2)	0.0426 (7)	
C11	0.1688 (4)	0.9204 (3)	0.2526 (3)	0.0532 (10)	
H11A	0.1894	0.9335	0.3173	0.064*	
H11B	0.2544	0.9263	0.2264	0.064*	
C9	0.0500 (4)	0.7490 (3)	0.3036 (3)	0.0563 (11)	
H9	0.0417	0.7855	0.3579	0.068*	
C5	0.0120 (4)	0.5898 (3)	0.2097 (3)	0.0537 (11)	
C10	0.0009 (4)	0.6417 (4)	0.2903 (3)	0.0620 (11)	
H10	−0.0392	0.6051	0.3355	0.074*	
C2	0.4878 (3)	0.7516 (3)	0.0400 (2)	0.0401 (8)	
C14	−0.1102 (5)	1.1646 (3)	0.1387 (3)	0.0698 (13)	
H14	−0.1726	1.2181	0.1132	0.084*	
C4	0.7257 (4)	0.8173 (3)	0.0722 (3)	0.0501 (9)	
C15	0.0006 (5)	1.1360 (4)	0.0964 (3)	0.0710 (13)	
H15	0.0142	1.1702	0.0420	0.085*	
C16	0.0911 (4)	1.0574 (3)	0.1337 (3)	0.0586 (11)	
H16	0.1666	1.0380	0.1049	0.070*	
C12	−0.0375 (4)	1.0353 (3)	0.2544 (3)	0.0546 (10)	
H12	−0.0503	0.9998	0.3084	0.066*	
C6	0.0723 (4)	0.6405 (3)	0.1425 (3)	0.0622 (11)	
H6	0.0794	0.6036	0.0881	0.075*	
N1	0.8110 (4)	0.8818 (3)	0.0910 (3)	0.0770 (11)	
C13	−0.1290 (4)	1.1141 (4)	0.2192 (3)	0.0632 (12)	
H13	−0.2034	1.1335	0.2491	0.076*	
C7	0.1227 (4)	0.7476 (3)	0.1569 (3)	0.0566 (11)	
H7	0.1649	0.7828	0.1120	0.068*	

### Atomic displacement parameters (Å^2^)

**Table:**

	*U*^11^	*U*^22^	*U*^33^	*U*^12^	*U*^13^	*U*^23^
Ni1	0.0332 (3)	0.0344 (4)	0.0365 (4)	0.0045 (3)	0.0013 (3)	−0.0016 (3)
Br1	0.0699 (4)	0.0431 (3)	0.1335 (5)	−0.0074 (2)	−0.0270 (3)	−0.0002 (3)
S1	0.0350 (5)	0.0405 (5)	0.0563 (6)	0.0044 (4)	−0.0003 (4)	−0.0029 (5)
S2	0.0349 (5)	0.0382 (5)	0.0699 (7)	0.0047 (4)	0.0047 (5)	−0.0076 (5)
C3	0.040 (2)	0.044 (2)	0.057 (2)	−0.0019 (18)	0.0059 (18)	−0.004 (2)
N2	0.063 (2)	0.048 (2)	0.086 (3)	0.0080 (18)	0.013 (2)	−0.010 (2)
C8	0.037 (2)	0.040 (2)	0.052 (2)	0.0020 (16)	−0.0039 (17)	0.000 (2)
C1	0.041 (2)	0.038 (2)	0.039 (2)	−0.0011 (16)	0.0011 (16)	−0.0029 (16)
N3	0.0439 (18)	0.0342 (17)	0.0487 (18)	−0.0015 (14)	0.0017 (14)	−0.0087 (16)
C11	0.048 (2)	0.047 (2)	0.061 (3)	−0.0023 (18)	−0.0080 (19)	−0.002 (2)
C9	0.069 (3)	0.049 (3)	0.048 (2)	−0.002 (2)	−0.001 (2)	−0.002 (2)
C5	0.038 (2)	0.039 (2)	0.079 (3)	0.0039 (17)	−0.014 (2)	0.005 (2)
C10	0.069 (3)	0.053 (3)	0.063 (3)	−0.004 (2)	0.005 (2)	0.014 (2)
C2	0.043 (2)	0.038 (2)	0.0396 (19)	0.0048 (16)	0.0044 (15)	−0.0023 (17)
C14	0.071 (3)	0.039 (2)	0.092 (4)	0.010 (2)	−0.020 (3)	−0.003 (3)
C4	0.047 (2)	0.045 (2)	0.056 (2)	0.004 (2)	0.0011 (19)	−0.005 (2)
C15	0.086 (3)	0.053 (3)	0.075 (3)	0.009 (3)	0.010 (3)	0.013 (2)
C16	0.066 (3)	0.049 (3)	0.065 (3)	−0.003 (2)	0.025 (2)	0.005 (2)
C12	0.054 (3)	0.061 (3)	0.048 (2)	−0.003 (2)	0.005 (2)	−0.009 (2)
C6	0.063 (3)	0.055 (3)	0.069 (3)	−0.003 (2)	0.012 (2)	−0.017 (2)
N1	0.059 (2)	0.064 (3)	0.103 (3)	−0.011 (2)	−0.011 (2)	−0.016 (2)
C13	0.052 (3)	0.062 (3)	0.074 (3)	0.009 (2)	0.002 (2)	−0.020 (3)
C7	0.056 (2)	0.050 (3)	0.065 (3)	−0.005 (2)	0.016 (2)	−0.004 (2)

### Geometric parameters (Å, º)

**Table:**

Ni1—S2^i^	2.1642 (9)	C9—C10	1.376 (5)
Ni1—S2	2.1642 (9)	C9—H9	0.9300
Ni1—S1	2.1773 (9)	C5—C10	1.366 (6)
Ni1—S1^i^	2.1773 (9)	C5—C6	1.366 (6)
Br1—C5	1.908 (4)	C10—H10	0.9300
S1—C1	1.740 (3)	C14—C15	1.366 (6)
S2—C2	1.737 (3)	C14—C13	1.373 (6)
C3—N2	1.146 (4)	C14—H14	0.9300
C3—C2	1.433 (5)	C4—N1	1.144 (5)
C8—C7	1.379 (5)	C15—C16	1.360 (6)
C8—C9	1.385 (5)	C15—H15	0.9300
C8—C11	1.517 (5)	C16—H16	0.9300
C1—C2	1.360 (5)	C12—C13	1.358 (6)
C1—C4	1.427 (5)	C12—H12	0.9300
N3—C16	1.343 (5)	C6—C7	1.379 (5)
N3—C12	1.346 (5)	C6—H6	0.9300
N3—C11	1.487 (4)	C13—H13	0.9300
C11—H11A	0.9700	C7—H7	0.9300
C11—H11B	0.9700		
			
S2^i^—Ni1—S2	180.00 (5)	C6—C5—Br1	118.9 (3)
S2^i^—Ni1—S1	87.84 (4)	C5—C10—C9	119.0 (4)
S2—Ni1—S1	92.16 (4)	C5—C10—H10	120.5
S2^i^—Ni1—S1^i^	92.16 (4)	C9—C10—H10	120.5
S2—Ni1—S1^i^	87.84 (4)	C1—C2—C3	123.0 (3)
S1—Ni1—S1^i^	180.0	C1—C2—S2	120.8 (3)
C1—S1—Ni1	103.21 (11)	C3—C2—S2	116.2 (3)
C2—S2—Ni1	103.42 (12)	C15—C14—C13	119.6 (4)
N2—C3—C2	175.7 (4)	C15—C14—H14	120.2
C7—C8—C9	118.7 (4)	C13—C14—H14	120.2
C7—C8—C11	120.7 (3)	N1—C4—C1	176.5 (4)
C9—C8—C11	120.6 (3)	C16—C15—C14	119.9 (4)
C2—C1—C4	122.6 (3)	C16—C15—H15	120.0
C2—C1—S1	120.3 (3)	C14—C15—H15	120.0
C4—C1—S1	117.1 (2)	N3—C16—C15	120.2 (4)
C16—N3—C12	120.4 (3)	N3—C16—H16	119.9
C16—N3—C11	120.4 (3)	C15—C16—H16	119.9
C12—N3—C11	119.2 (3)	N3—C12—C13	120.9 (4)
N3—C11—C8	112.6 (3)	N3—C12—H12	119.6
N3—C11—H11A	109.1	C13—C12—H12	119.6
C8—C11—H11A	109.1	C5—C6—C7	118.5 (4)
N3—C11—H11B	109.1	C5—C6—H6	120.7
C8—C11—H11B	109.1	C7—C6—H6	120.7
H11A—C11—H11B	107.8	C12—C13—C14	119.0 (4)
C10—C9—C8	120.7 (4)	C12—C13—H13	120.5
C10—C9—H9	119.7	C14—C13—H13	120.5
C8—C9—H9	119.7	C8—C7—C6	121.1 (4)
C10—C5—C6	122.0 (4)	C8—C7—H7	119.4
C10—C5—Br1	119.1 (3)	C6—C7—H7	119.4

Symmetry code: (i) −*x*+1, −*y*+1, −*z*.

### Hydrogen-bond geometry (Å, º)

**Table:**

*D*—H···*A*	*D*—H	H···*A*	*D*···*A*	*D*—H···*A*
C16—H16···N2	0.93	2.49	3.384 (6)	162
