# Supplementary figures and images for: Crystal structure of bis­[1-(4-bromo­benz­yl)pyridinium] bis­(1,2-di­cyano­ethene-1,2-di­thiol­ato-κ2 S,S′)nickelate(II)
Source: Acta Crystallogr Sect E Struct Rep Online. 2014 Nov 12;70(Pt 12):m395–6. doi: 10.1107/S1600536814024222 (PMC4257415; doi:10.1107/S1600536814024222)

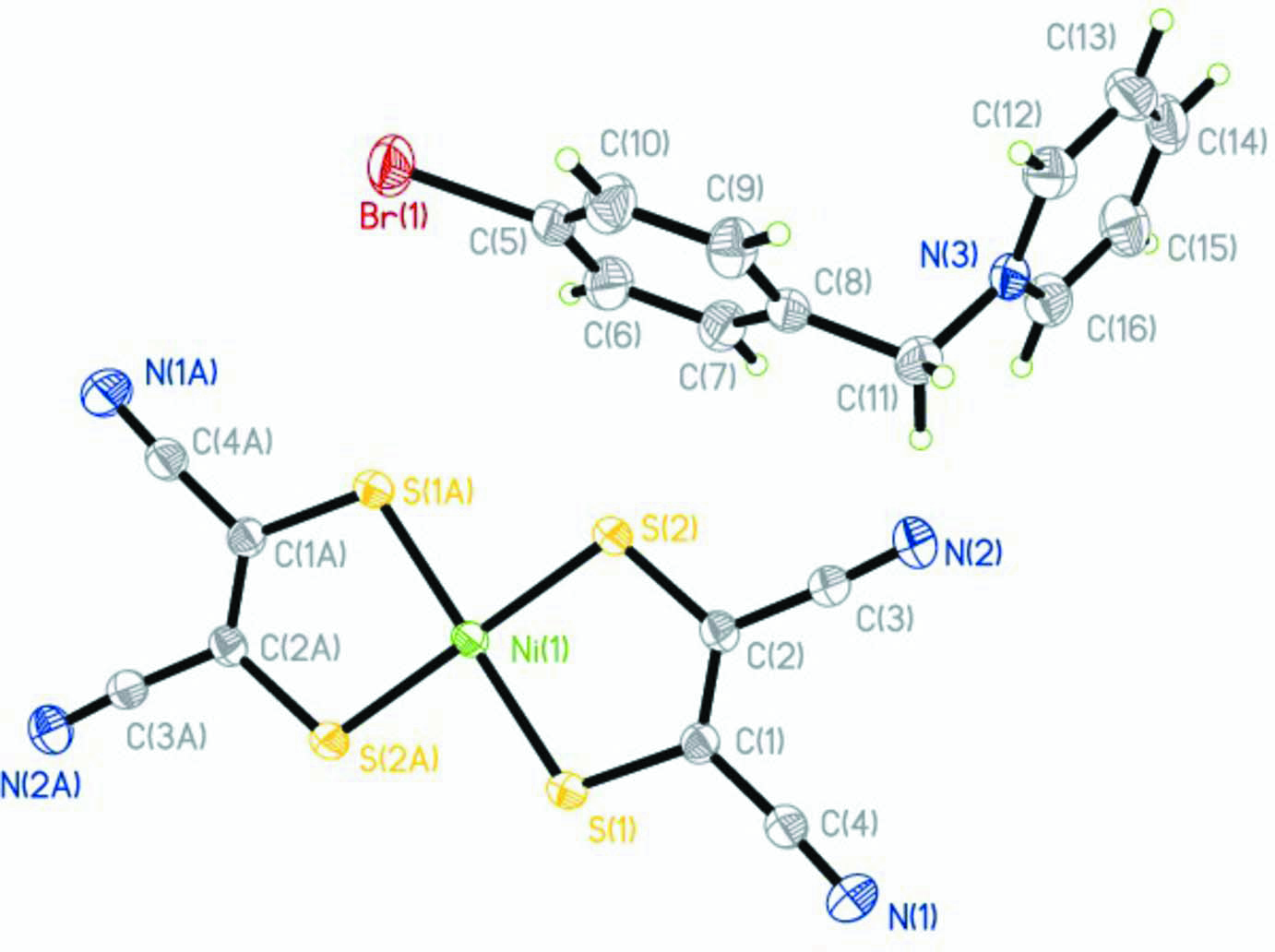

Supplement: Supplementary file 3 [file e-70-0m395-fig1.tif]

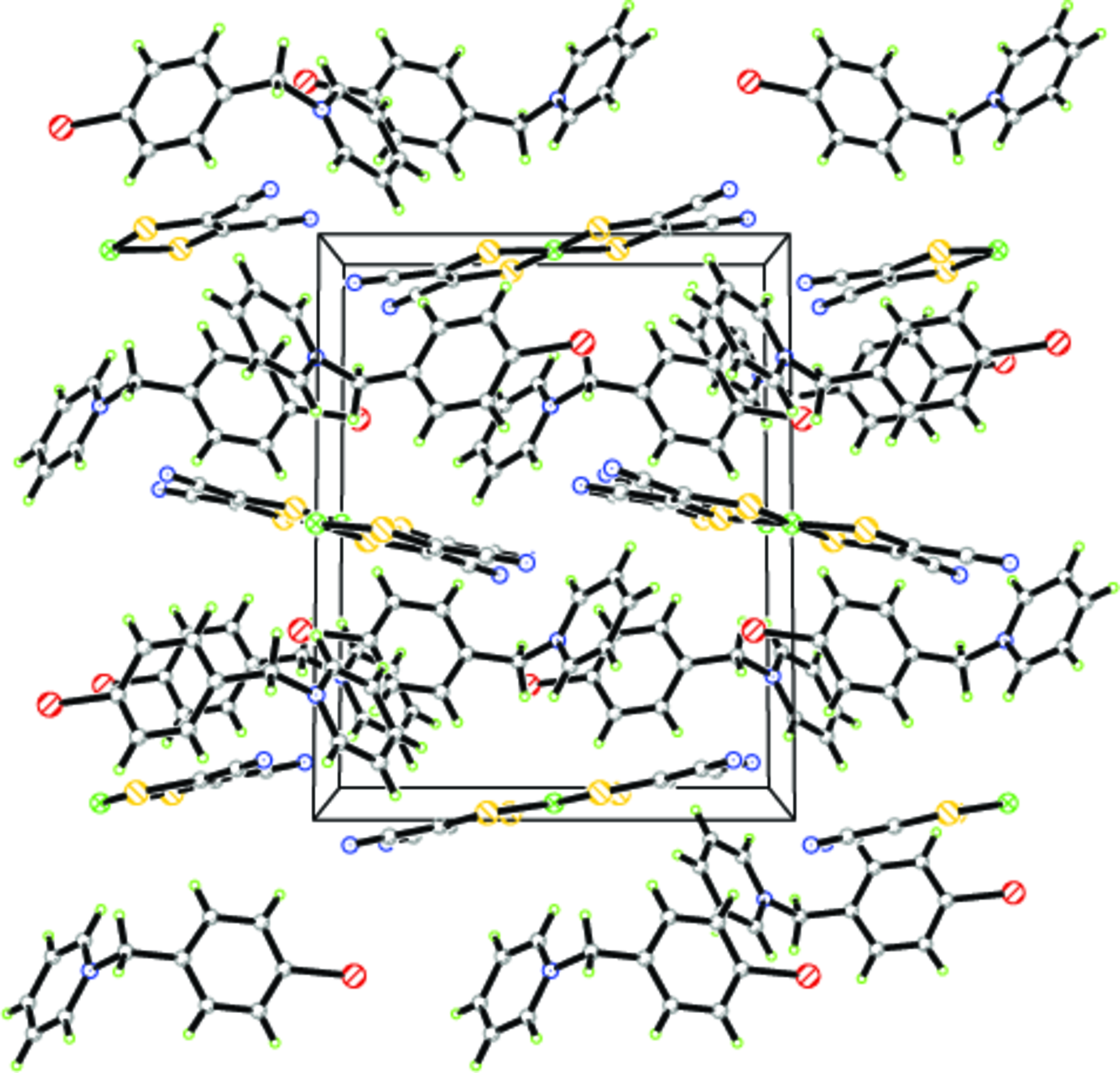

Supplement: Supplementary file 4 [file e-70-0m395-fig2.tif]
